# Surgical treatment of symptomatic pineal cysts without hydrocephalus—meta-analysis of the published literature

**DOI:** 10.1007/s00701-021-05054-0

**Published:** 2021-12-02

**Authors:** Riccardo Masina, Ali Ansaripour, Vladimír Beneš, Moncef Berhouma, Joham Choque-Velasquez, Per Kristian Eide, Stepan Fedorko, Steffen Fleck, Juha Hernesniemi, Andrzej Koziarski, Martin Májovský, Andrzej Podgorski, Henry Schroeder, Charles Teo, Andreas W. Unterberg, Jacky T. Yeung, Angelos Kolias, Thomas Santarius

**Affiliations:** 1 Department of Neurosurgery, Addenbrooke’s Hospital, University of Cambridge, Cambridge, UK; 2 Department of Neurosurgery and Neurooncology, 1St Faculty of Medicine, Charles University, Military University Hospital, U Vojenske Nemocnice, 1200 Prague 6, Czech Republic; 3 Department of Neurosurgery B, Pierre-Wertheimer Neurological and Neurosurgical Hospital, Hospices Civils de Lyon, 59, Boulevard Pinel, 69394 Lyon Cedex 03, France; 4 Department of Neurosurgery, Helsinki University Hospital, University of Helsinki, Helsinki, Finland; 5 Department of Neurosurgery, University of Heidelberg, Heidelberg, Germany; 6 Department of Neurosurgery, Oslo University Hospital-Rikshospitalet, Nydalen, Postboks 4950, 0424 Oslo, Norway; 7 Faculty of Medicine, University of Oslo, Oslo, Norway; 8 Institute of Clinical Medicine, Faculty of Medicine, University of Oslo, Oslo, Norway; 9 Department of Neurosurgery, Cairo University, Cairo, Egypt; 10 Juha Hernesniemi International Center for Neurosurgery, Henan Provincial People’s Hospital, Zhengzhou, China; 11 Department of Neurosurgery, Military Institute of Medicine, Warsaw, Poland; 12 Department of Neurosurgery, Greifswald University Medicine, Greifswald, Germany; 13 Centre for Minimally Invasive Neurosurgery, Prince of Wales Hospital, Sydney, NSW Australia; 14 Department of Clinical Neurosciences, University of Cambridge, Cambridge, UK; 15 Department of Physiology, Development, and Neuroscience, University of Cambridge, Cambridge, UK

**Keywords:** Pineal, Pineal cyst, Symptomatic pineal cyst, Non-hydrocephalic symptomatic pineal cyst, Hydrocephalus, Headache

## Abstract

**Background:**

To examine published data and assess evidence relating to safety and efficacy of surgical management of symptomatic pineal cysts without hydrocephalus (nhSPC), we performed a systematic review of the literature and meta-analysis.

**Methods:**

Following the PRISMA guidelines, we searched Pubmed and SCOPUS for all reports with the query ‘Pineal Cyst’ AND ‘Surgery’ as of March 2021, without constraints on study design, publication year or status (PROSPERO_CRD:42,021,242,517). Assessment of 1537 hits identified 26 reports that met inclusion and exclusion criteria.

**Results:**

All 26 input studies were either case reports or single-centre retrospective cohorts. The majority of outcome data were derived from routine physician-recorded notes. A total of 294 patients with surgically managed nhSPC were identified. Demographics: Mean age was 29 (range: 4–63) with 77% females. Mean cyst size was 15 mm (5–35). Supracerebellar-infratentorial approach was adopted in 90% of cases, occipital-transtentorial in 9%, and was not reported in 1%. Most patients were managed by cyst resection (96%), and the remainder by fenestration. Mean post-operative follow-up was 35 months (0–228). Presentation: Headache was the commonest symptom (87%), followed by visual (54%), nausea/vomit (34%) and vertigo/dizziness (31%). Other symptoms included focal neurology (25%), sleep disturbance (17%), cognitive impairment (16%), loss of consciousness (11%), gait disturbance (11%), fatigue (10%), ‘psychiatric’ (2%) and seizures (1%). Mean number of symptoms reported at presentation was 3 (0–9). Outcomes: Improvement rate was 93% (to minimise reporting bias only consecutive cases from cohort studies were considered, *N* = 280) and was independent of presentation. Predictors of better outcomes were large cyst size (OR = 5.76; 95% CI: 1.74–19.02) and resection over fenestration (OR = 12.64; 3.07–52.01). Age predicted worse outcomes (OR = 0.95; 0.91–0.99). Overall complication rate was 17% and this was independent of any patient characteristics. Complications with long-term consequences occurred in 10 cases (3.6%): visual disturbance (3), chronic incisional pain (2), sensory disturbance (1), fatigue (1), cervicalgia (1), cerebellar stroke (1) and mortality due to myocardial infarction (1).

**Conclusions:**

Although the results support the role of surgery in the management of nhSPCs, they have to be interpreted with a great deal of caution as the current evidence is limited, consisting only of case reports and retrospective surgical series. Inherent to such studies are inhomogeneity and incompleteness of data, selection bias and bias related to assessment of outcome carried out by the treating surgeon in the majority of cases. Prospective studies with patient-reported and objective outcome assessment are needed to provide higher level of evidence.

**Supplementary Information:**

The online version contains supplementary material available at 10.1007/s00701-021-05054-0.

## Introduction

Pineal cysts (PCs) are benign, non-cancerous cysts arising from the pineal gland. They are relatively common as they can be identified on approximately 0.5–5% of the brain MRI scans [17, 18, 29, 33] and 20% of autopsies [20, 44]. Most PCs are small (< 10 mm) and asymptomatic, but some cause symptoms.

The most widely adopted management of patients with PCs is to first make sure that the cyst is non-neoplastic [33]. Management of SPCs with hydrocephalus is well-established [21]. Acute hydrocephalic SPC cases have traditionally been treated with shunts and more recently with endoscopic third ventriculostomy (ETV) with or without biopsy/fenestration [21], although stereotactic aspiration, resection and conservative management have been used in some cases [2, 16, 32, 36, 45].

The diagnostic entity of nhSPC is in itself controversial and a consensus of diagnostic criteria is currently lacking. Likewise, management of non-hydrocephalic SPCs (nhSPCs) is subject of controversy [27]. Presentation usually consists of chronic headaches, visual and other symptoms in the presence of a PC and absence of ventriculomegaly on imaging. It is currently not possible to determine the exact incidence of nhSPCs, not least because of lack of familiarity with this diagnosis among physicians. As a result, patients’ symptoms are usually disregarded, and nhSPCs are considered incidental findings. Indeed, the indication for MRI scan in ‘incidental PCs’ reported in epidemiological studies were, in fact, symptoms classically associated with nhSPC, i.e. headache and gaze paresis, in at least 50–75% of cases [1, 17, 33, 42]. Similarly, headache was found significantly more commonly in patients with ‘incidental PCs’ than in a matched control group [40].

Until the first large cohort of patients with nhSPCs published by Kalani et al. in 2015 [23], there were only case reports suggesting a tenuous relationship between cyst resection and improvement of symptoms and, as such, nhSPC did not exist as a neurosurgical entity. Since then, several clinical series reported similar results [8, 12–14, 24, 28, 46]. Recent systematic reviews of headache in adult [31] and overall outcome in paediatric patients [6] reported a high level of safety and success in controlling symptoms.

Nevertheless, a great deal of uncertainty exists about the role of surgery in the management of nhSPC. Hence, we set to carry out a rigorous systematic review and meta-analysis of all available literature to determine the safety and efficacy of the surgical management of nhSPCs. Our secondary objectives were to study the demographics and presentations of nhSPCs and their relationship with clinical outcomes.

## Materials and methods

**Fig. 1:**
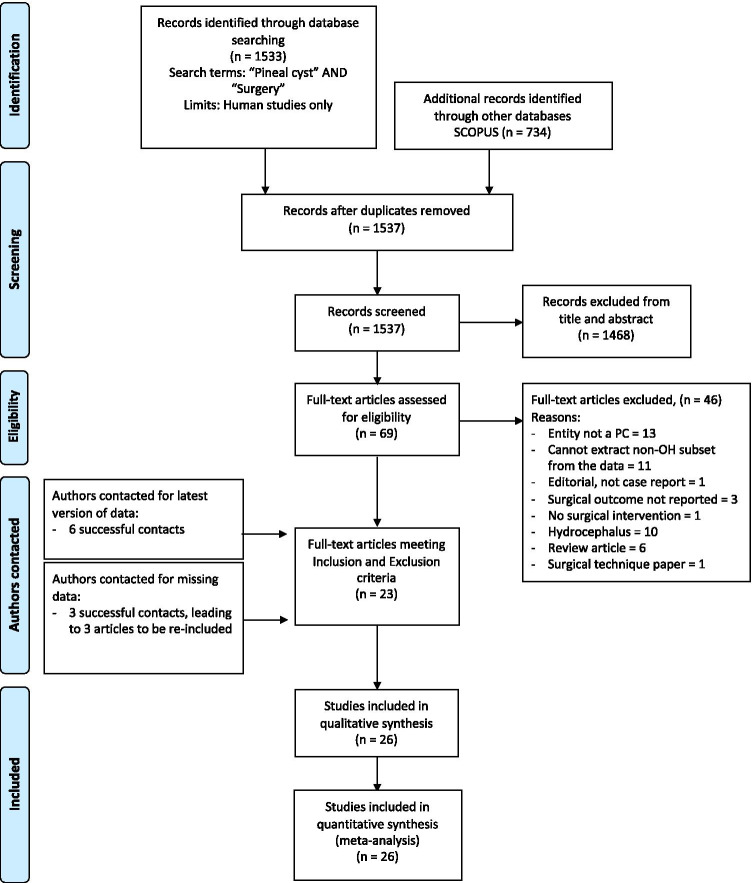
Prisma flow diagram summarising the systematic review algorithm

This systematic review was constructed in accordance with PRISMA guidelines and is registered in the PROSPERO prospective register of systematic review (PROSPERO ID: CRD42021242517). The selection criteria, search, data extraction and verification processes are summarised in Fig. 1 and Table 1. The complete list of extracted parameters is summarised in Supplementary Table 1.

**Table 1:** Inclusion and exclusion criteria according to the PICOS (Population, Intervention, Control, Outcomes, Study) framework

**Population:**	**Inclusion:** human studies; all demographics
	**Exclusion:** ventriculomegaly; hydrocephalus; additional CNS pathology
Intervention:	Surgery (resection and fenestration)
Control:	No control available
Outcome:	Efficacy: % of patients whose condition has been reported as improved post-operatively
	Safety: % of patients who experienced complications, both short-term and long-term
Study:	All study designs accepted

### Objectives

The primary objective of this review was to measure safety and efficacy of neurosurgical treatment by cyst resection or fenestration of symptomatic patients with PC without hydrocephalus.

Secondary objectives included outcome comparison between surgical approaches, subgroup analysis by demographic characteristics such as gender and age-group and examination of pre-operative clinical and radiological variables in relation to outcome.

### Eligibility criteria

According to prospectively deposited eligibility criteria, we included any reports of patients with surgically managed symptomatic PCs without hydrocephalus, which in routine clinical practice equates to radiologically confirmed ventriculomegaly. We excluded any patient with additional co-existing CNS pathology (e.g. brain tumours).

### Search strategy

Search strategy combined relevant medical subject headings (MeSH) and keywords. The search strategy has been drafted by RM and reviewed by TS—it is available in Supplementary Table 2.

Reports had to be searchable through the PubMed and SCOPUS databases via the search terms ‘Pineal Cyst’ and ‘Surgery’ as of March 2021. No constraints on study design, year of publication or publication status were imposed. From the 1537 unique records identified by this search, screening for relevance by title and abstract resulted in 1468 articles being excluded. Of the remaining 69 articles selected for full-text evaluation, a total of 46 were excluded (Fig. 1) for the following reasons: pineal lesion not a pineal cyst (13); the study not allowing to extract the cases without hydrocephalus from the reported cohort (11); radiologically confirmed hydrocephalus (10); the study being a review article (6); surgical outcome not reported (3); report of surgical technique only (1) and no surgical intervention (1); the report being an editorial letter.

The authors of all case series with *N* > 5 published since 2000 were successfully contacted for the latest version of their data. As a result, the authors from 3 studies that were excluded because of missing data were able to provide the necessary information, which resulted in their work meeting all inclusion and exclusion criteria. Figure 1 provides a summary of this study selection process.

### Data extraction

The final dataset consisted of 26 studies and was analysed by two independent reviewers to extract a pre-determined set of study-specific, patient-specific, surgery-specific and outcome-specific parameters to be used for all subsequent analysis (Supplementary Table 1). All instances of discrepancy were resolved by discussion leading to a mutually agreed consensus. Following data extraction, all contacted authors were asked to confirm the accuracy of the dataset, with particular focus on complications and improvement rates.

### Symptoms

Given that we were dealing with multiple retrospective studies of a relatively rare condition whose understanding has been evolving over last few decades, there is, understandably, a variation in the recognition, description and categorisation of symptoms. We adopted a pragmatic approach by pooling study-specific symptoms into categories listed in Table 2.

**Table 2:** Summary of criteria adopted for pooling symptoms for the analysis

Label	Symptoms included
Headache	Headache; head fullness; pressure in the head; migraines
Visual_sx	Blurred vision; double vision; limitation of upward gaze/convergence; Parinaud syndrome; visual field defects
Nausea_vomit	Nausea; vomiting
Vertigo_dizziness	Vertigo; dizziness
Neurology_NOS	Paraesthesia; paresis; dysphasia/dysarthria; tremor
Sleep	Sleep disturbance
Cognitive	Memory deficit; attention deficit; cognitive impairment; ‘brain fog’; concentration deficit; confusion
Transient_LoC	Syncope; fainting; drop attacks
Seizures	Seizures
Gait	Ataxia; gait instability
Fatigue	Fatigue; malaise
Psych_depression	Depression; anxiety; ‘functional neurological disorder’; personality changes

### Indication for surgery and surgical approaches

**Fig. 2:**
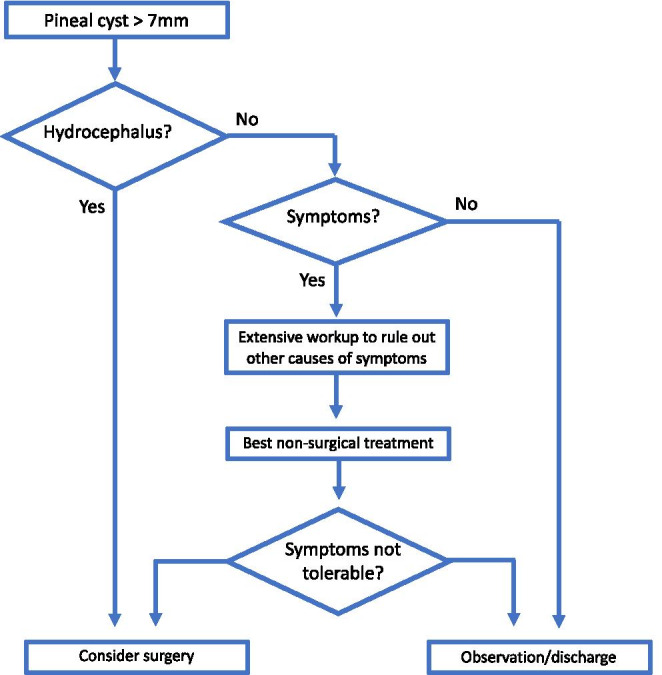
Flow-chart summarising the main decisions taken in the management of patients with nhSPC. This is broadly representative of the contributing series, but individual surgeons have developed their own specific management algorithms—see individual series for details [4, 8, 13, 14, 23, 24, 28, 46]

The indication for surgery was broadly consistent with the algorithm published by Majovsky et al. in 2017 [28] (Fig. 2). Two principal surgical approaches were used: supracerebellar infratentorial (SCIT) and occipital transtentorial (OTT). The surgical management of cyst was classified as either resection or fenestration, as it was not possible to determine the exact degree of resection.

### Outcome

We have taken a practical approach and used a definition that is unambiguously applicable to all cases in this cohort and defined ‘overall improvement’ as overall less symptom-related burden, where both pre-existing and any newly acquired symptoms following surgery were considered. In reality, ‘overall improvement’ is probably closer to the intuitive and, realistically, most important to patients ‘feeling overall better’ or ‘having better quality of life’. Definitions and methods of determining of ‘overall improvement’ in individual studies are listed in Supplementary Table 1.

Complications were included as listed in the selected papers. ‘Transient complication’ was a complication that was not present at the latest follow-up.

Overall improvement and complication rates were estimated from the subset of the cohort extracted from consecutive case series only (*N* = 280). This is to minimise the effect of reporting bias, e.g. it is more likely that cases with favourable than unfavourable were reported. In contrast, all cases (*N* = 294) were included in the summary to make reader aware of all reported complications. Reporting bias was assessed using funnel plots (Supplementary Fig. 1). Objective assessment of study characteristics and risk of bias was done according to NHLBI Study Quality Assessment Tool [43] (Supplementary Fig. 2).

The associations between (1) presenting symptoms and outcomes; (2) patient-specific characteristics and outcomes; (3) surgery-specific characteristics and outcomes were assessed using univariate logistic regression models. Possible interactions between covariates are explored in multivariate regression models. The strength of each association is expressed as odds ratio with Wald 95% confidence intervals. Simple comparisons between groups were performed using the appropriate test statistics (Student’s *t* test, Chi-squared test, Fisher’s exact test). *p* values are reported as unadjusted, and the conventional threshold of 0.05 has been used for statistical significance. *R* was used to perform every part of the analysis as well as to generate all summary plots.

## Results

A full description of the study selection process and results is provided in the Methods section and summarised in Fig. 1.

### Study characteristics

Table 3 provides a summary of the characteristics of the 26 articles that were included for the qualitative and quantitative analysis. There were 18 case series and 8 case reports, all published between 1989 and 2021. No case–control, prospective cohort nor interventional studies had been published as of March 2021.

**Table 3:** Summary of reports that met inclusion and exclusion criteria

Year	Journal	1st author	Last author	Type of study	*N*
2021/2015	J Neurosurg	Yeung/Kalani	Teo	Case series	80
2017	World Neurosurg	Majovsky	Benes	Case series	20
2017	Acta Neurochir	Eide	Ringstad	Case series	21
2019	World Neurosurg	El Damaty	Schroeder	Case series	43
2019	Br J Neurosurg	Koziarski	Zielinski	Case series	28
2018	JNS	Fedorko	Unterberg	Case series	7
2019	Surg Neurol Int	Choque-Velasquez	Hernesniemi	Case series	44
2008	Pediatr Neurosurg	Morgan	Schneider	Case report	1
2002	Acta Neurochir	Michielsen	Caemaert	Case series	2
2013	Neurochirurgie	Berhouma	Vallee	Case series	6
2011	Minim Invas Neurosurg	Menovsky	Grotenhuis	Case report as letter to editor	1
1992	J Neurosurg	Wisoff	Epstein	Case series	4
1997	Ann Diagn Pathol	Mena	Rushing	Case series	6
1989	J Neurol Neurosurg	Klein	Rubinstein	Case series	4
1991	Acta Neurochir	Oeckler	Feiden	Case series	3
1994	AJNR	Fleege	Scheithauer	Case series	10
2003	Childs Nerv Syst	Mandera	Kluczewska	Case series	3
2007	J Child Neurol	Stevens	Sood	Case report	1
2012	Childs Nerv Syst	Kahilogullari	Di Rocco	Case report	1
2013	Acta Paediatr	Meyer	Kutschke	Letter to editor with case report	1
2008	Folia Neuropathol	Taraszewska	Czernicki	Case series	2
1992	Surg Neurol	Miyatake	Nakashima	Case report	1
2014	Neurosurg Rev	Thaher	Hopf	Case report	1
2008	Neurosurgery	Gore	Nakaji	Case report	1
2020	J Clin Neurosci	Tanaka	Litofsky	Case series	3
					***N* = 294**

According to the NHLBI Study Quality Assessment Tool, overall study quality was reported as ‘fair’ or above for all 26 studies (Supplementary Fig. 2). Through collaboration with the authors of the majority of the published case series, we were able to acquire, review and analyse raw data of 261 out of the 294 cases.

### Cohort characteristics—demographics

**Fig. 3:**
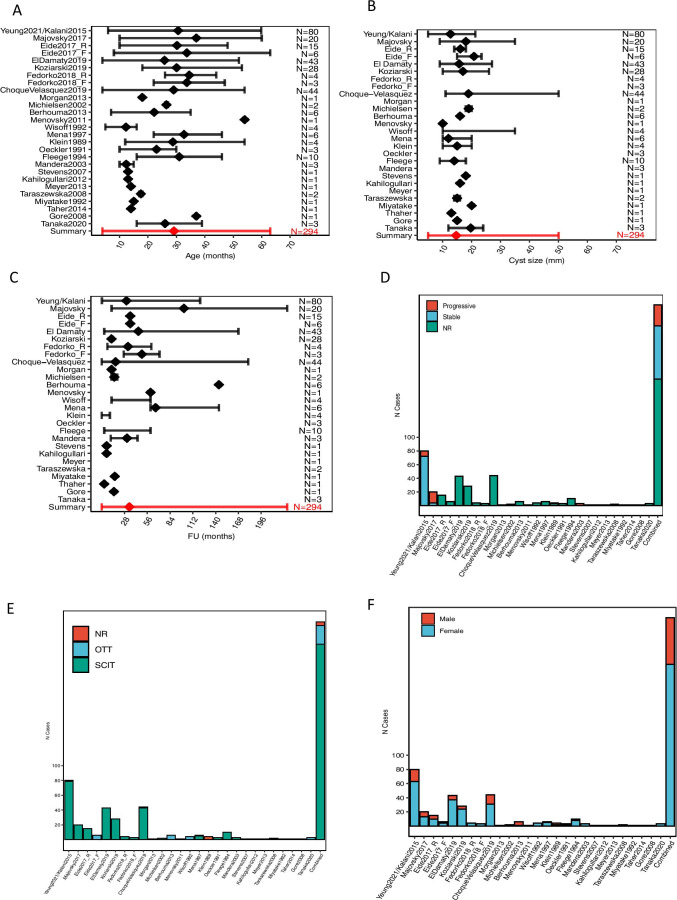
Summary of cohort characteristics. **A** Age at the time of operation. **B** Cyst size at the time of operation. **C** Duration of post-operative follow-up. **D** Symptom progression pre-operatively. **E** Surgical approach. **F** Gender at the time of operation. Forest plots report mean (diamonds) ± max/min (error bars). The absence of a diamond or error bars indicate that the information was not available for that study. In all graphs, the final entry represents a summary of all the available data. Yeung2021/Kalani2015 have been grouped together as Kalani’s case series [23] is fully contained within Yeung’s case series [46]. The figure is available in colour online. SCIT, SupraCerebellar Infra-Tentorial approach; OTT, occipital trans-tentorial approach; NR, not reported

A total of 294 patients were identified who underwent surgery for nhSPC. A summary of the characteristics of this cohort is available in Fig. 3 and Supplementary Table 1 and Supplementary Table 3. Mean age was 29.3 years (3–63), and 18.7% (55/294) of patients were under 18 years of age. Female patients predominated 77% (228/294). Mean cyst size was 15 mm (5–35 mm). The mean length of post-operative follow-up was 34.6 months (0–228). The supra-cerebellar infratentorial (SCIT) and occipital trans-tentorial (OTT) approaches were used in 90% and 9% of cases, respectively. Approach was not reported in 5 cases. Most patients were managed by cyst resection (96%) and only a few (4%) by fenestration.

### Cohort characteristics—presentation

**Fig. 4:**
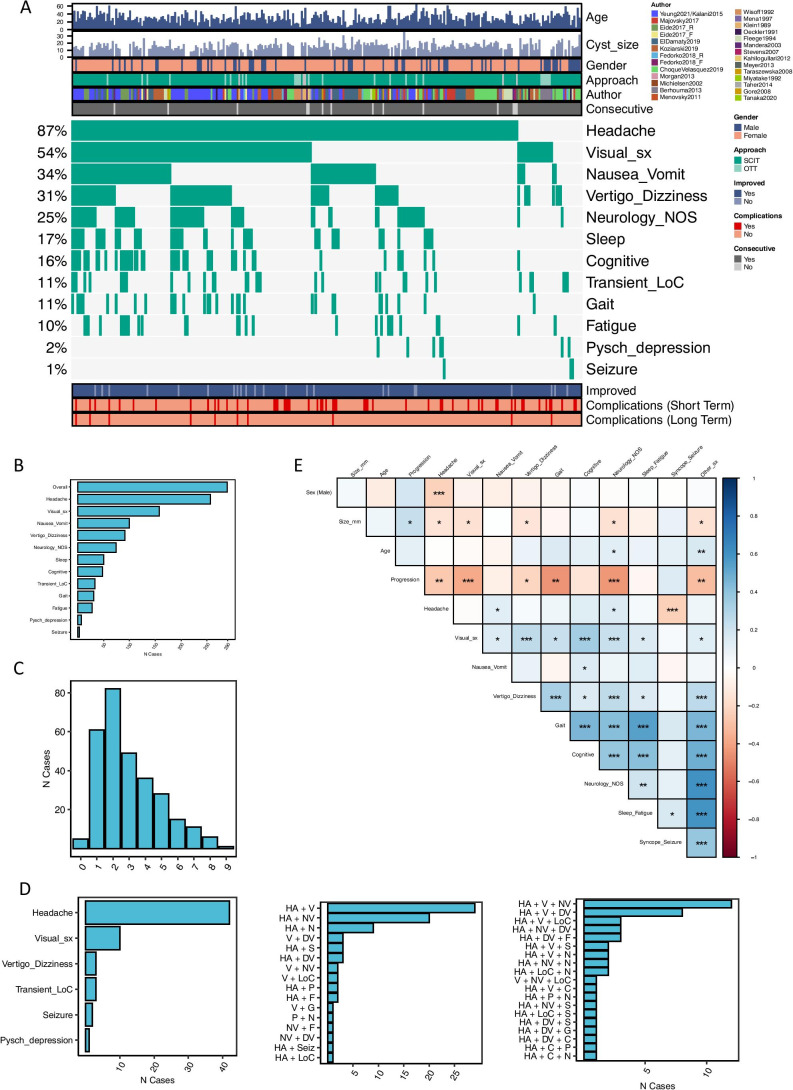
Summary of symptoms at presentation. **A** Summary of patient-specific demographic (top), presentation (middle) and outcome (bottom) features. Each patient is shown as a vertical segment along the *x* axis. Different features are labelled according to the legend. **B** Proportion of patients by presenting symptom. **C** Proportion of patients by number of symptoms at presentation. Of note, 5 patients reportedly had no symptoms at presentation. These are briefly described below. **D** Distribution of associated symptoms in patients presenting with only 1, 2 or 3 symptoms (from left to right, respectively). The distribution of patients presenting with 4 and 5 + symptoms is shown in Supplementary Fig. 5. **E** Co-occurrence of clinical characteristics at presentation. Data represented as correlation matrix, where the correlation coefficient (Pearson) between characteristics *X* and *Y* is shown on a colour scale ranging from blue (positive correlation) to red (negative correlation). White corresponds to a correlation coefficient of 0. Statistically significant correlations are marked by one or more ‘*’, according to a conventional notation of statistical significance (* < 0.05; ** < 0.01, *** < 0.005). *5 patients had no reported symptoms at presentation: (1) No symptom reported, surgical indication: ‘progressing cyst size’. (2) 4 years old asymptomatic, surgical indication: ‘cyst with partial solid enhancement’; (3) 54 years old asymptomatic, surgical indication: ‘solid posterior part of the cyst’; (4) 29 years old, no symptoms reported, surgical indication: ‘large cyst unspecified symptoms’; (5) 16 years old, presentation data not available, indication for surgery: not available. The figure is available in colour online. Size_mm_12: cyst size > 12 mm; Visual_sx, visual symptoms; Neurology_NOS, neurology not otherwise specified; Resection_extent, cyst resection, as opposed to cyst fenestration; Sleep, sleep disturbances; Pysch_depression, psychiatric symptoms of depression; Other_sx, any of the following symptoms: ‘Cognitive’, ‘Transient_LoC’, ‘Sleep’, ‘Pysch_depression’, ‘Seizure’, ‘Neurology_NOS’, ‘Fatigue’; LoC, loss of consciousness; HA, headache; V, visual symptoms; NV, nausea and vomiting; DV, dizziness and vertigo; P, psychiatric symptoms; F, fatigue; G, gait abnormalities; N, neurology not otherwise specified; Seiz, seizures; S, sleep disturbances

The types and frequencies of each presenting symptom are listed in Fig. 4. The mean number of presenting symptoms reported was 3 (range: 0–9). Mean pre-operative duration of symptom was 43.9 months (range: 0–492) (Fig. 3). Information about the pre-operative clinical course was available in 108 cases with 28% of cases were reported as progressive and 72% as stable (Fig. 3).

The prevalence of each combination of symptoms is shown in Fig. 4. The most common combination of two symptoms was headache and visual disturbance, followed by headache and nausea/vomiting.

Females are more likely to present with headaches than males, while seizures are more common in males. Pineal cyst size negatively correlates with headache, visual and vertigo presentations. Headaches correlate positively with sleep impairment, fatigue as well as neurological symptoms, and negatively correlate with seizures and transient impairment of consciousness. Visual symptoms strongly correlate with vertigo/dizziness, cognitive deficits and neurological symptoms and less strongly with nausea/vomiting, gait, sleep impairment and fatigue. Similarly, gait instability correlates strongly with cognitive, and vertigo and dizziness. Cognitive deficits are associated with sleep impairment and fatigue. They are also associated with neurological symptoms.

A complete summary of the strength of each association is available in Supplementary Table 4.

### Primary outcomes—efficacy

**Fig. 5:**
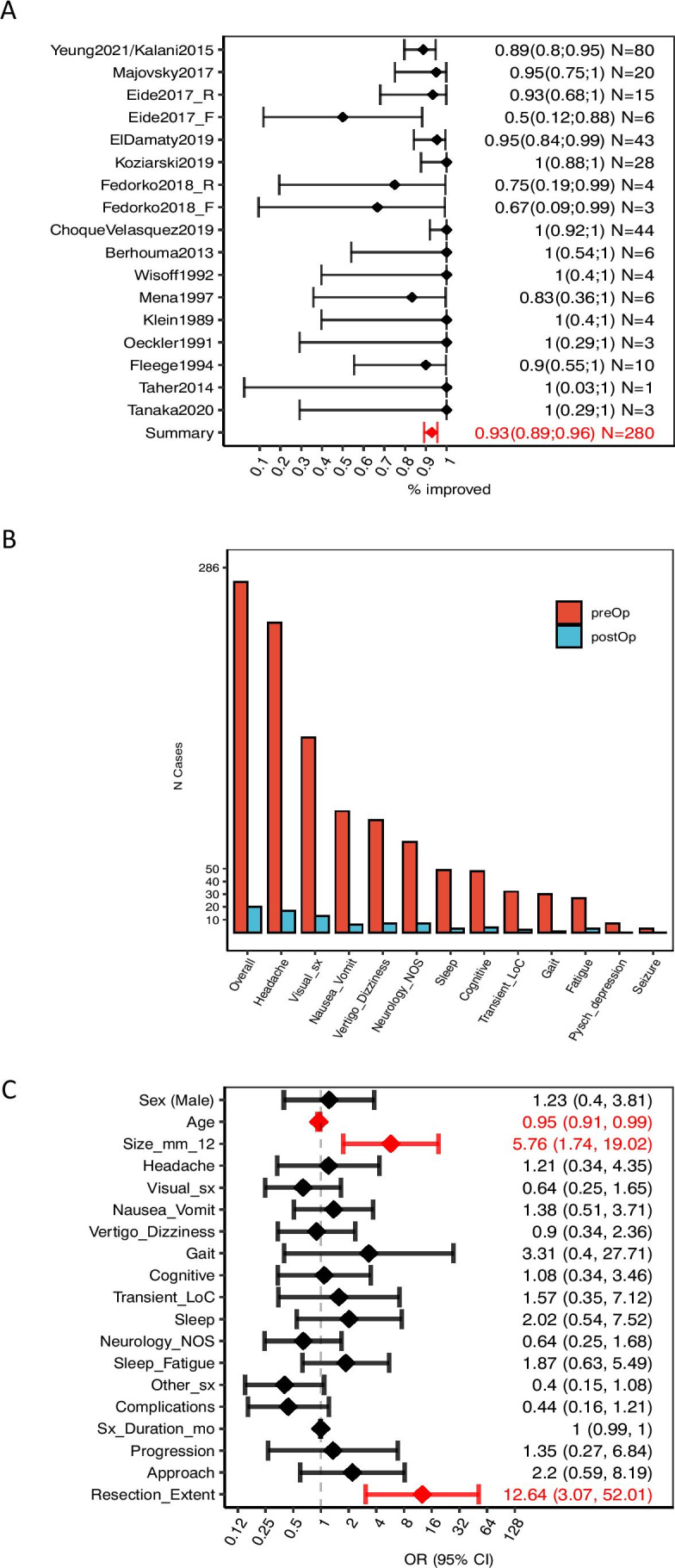
Summary of the efficacy profile of surgical management for nhSPC. **A** Improvement rate for each study and overall cohort. Data shown as mean (diamonds) ± 95% CI (error bars). Eide2017_R refers to the patients of Eide’s cohort that were managed by resection, while Eide2017_F refers to those managed by fenestration. Analogously, Fedorko2018_R refers to the patients in Fedorko’s series that were managed by resection, and Fedorko2018_F to those managed by fenestration. **B** Proportion of patients presenting with each symptom pre-operatively (red bars), and proportion of patients who did not improve post-operatively (blue bar). **C** Association between presenting characteristics and post-operative improvement, quantified by univariate logistic regression. Data shown as OR (diamonds) ± 95% CI. Statistically significant associations are shown in red. OR > 1 indicates that the characteristic is associated with better outcomes, while OR < 1 indicates an association with worse outcomes. Raw data is available as scatterplots in Supplementary Fig. 6. In all cases, improvement is defined as reduced symptom-related burden, where both pre-existing and any newly acquired symptoms following surgery are considered. Only data from consecutive case series has been included in the outcome analysis. The figure is available in colour online. Size_mm_12, cyst size > 12 mm; Visual_sx, visual symptoms; Neurology_NOS, neurology not otherwise specified; Resection_extent, cyst resection, as opposed to cyst fenestration; Sleep_fatigue: sleep disturbances or fatigue; Pysch_depression, psychiatric symptoms of depression; Other_sx, any of the following symptoms: ‘Cognitive’, ‘Transient_LoC’, ‘Sleep’, ‘Pysch_depression’, ‘Seizure’, ‘Neurology_NOS’, ‘Fatigue’; LoC, loss of consciousness

Of the 280 patients with nhSPC from consecutive case series, surgery resulted in overall improvement in 93% (Fig. 5). A comparison between pre- and post-operative symptoms for the entire cohort and for individual published papers are presented in Fig. 5 and Supplementary Fig. 3, respectively. Predictors of better outcomes were large cyst size (OR = 5.76; 95% CI: 1.74–19.02) and resection over fenestration (OR = 12.64; 3.07–52.01). Age predicted worse outcomes (OR = 0.95; 0.91–0.99).

### Primary outcomes—safety/complications

**Fig. 6:**
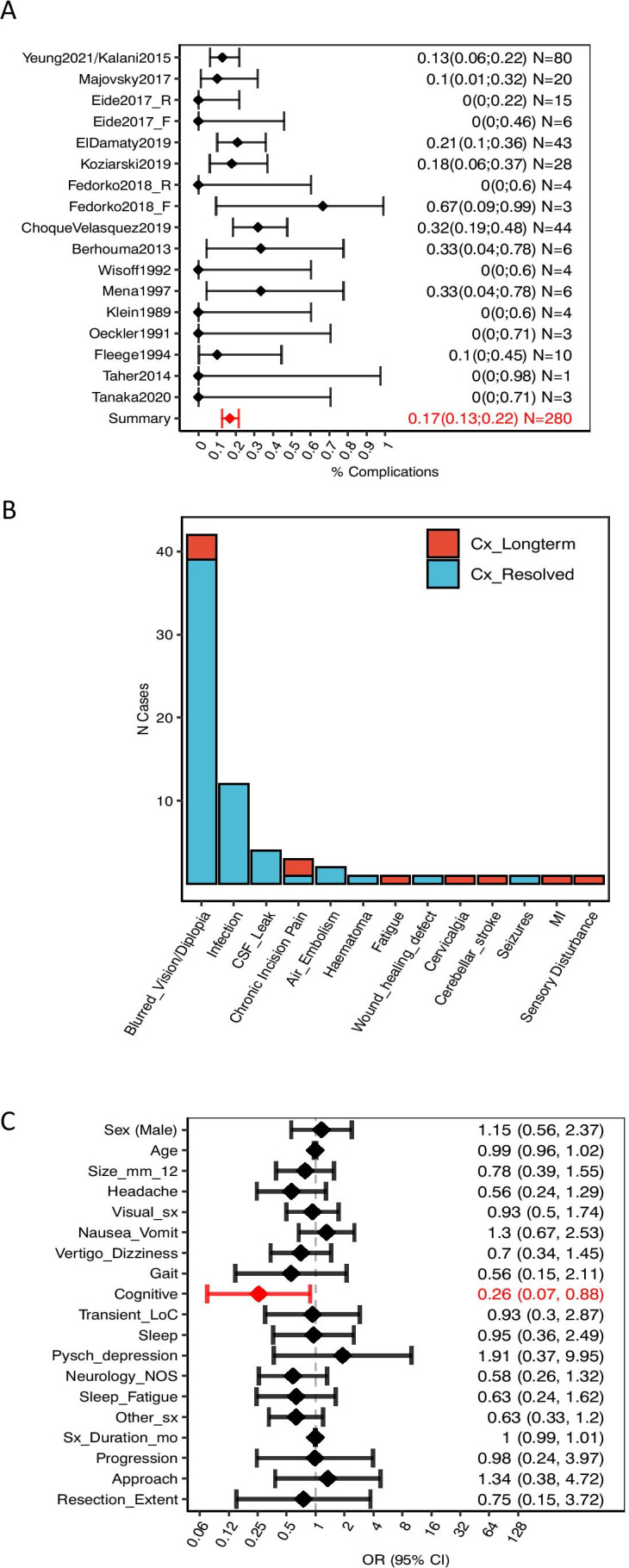
Summary of the safety profile of surgical management for nhSPC. **A** Complication rate for each study and overall cohort. As for ‘overall improvement’, complication rates were estimated from the subset of the cohort extracted from consecutive case series only (*N* = 277) to minimise the effect of reporting bias (see Methods). Data shown as mean (diamonds) ± 95% CI (error bars). Eide2017_R refers to the patients of Eide’s cohort that were managed by resection, while Eide2017_F refers to those managed by fenestration. Analogously, Fedorko2018_R refers to the patients in Fedorko’s series that were managed by resection, and Fedorko2018_F to those managed by fenestration. **B** Proportion of patients presenting with each complication. Here we included all cases (*N* = 294) to describe all reported complication that occurred during resection of nhSPCs. Complications that resolved by the last follow-up appointment are in blue, while those that persisted are in red. ‘Haematoma’ refers to an episode of bleeding into the 3rd ventricle that occurred in the early post-operative period in a 23-year-old female. EVD was placed for 7 days until the haematoma spontaneously resolved, and the patient had no neurological sequelae [28]. It is unclear whether the case of ‘Cerebellar stroke’ was merely a radiological finding or whether this was associated with clinical manifestations [15]. MI/mortality—mortality due to myocardial infarction [30]. ‘Chronic incisional pain’ refers to 3 cases reported by Yeung in which pain lasted beyond the peri-operative period. Of these, 2 patients underwent neuroma excision that resolved the pain, and 1 resolved spontaneously [29]. **C** Association between presenting characteristics and post-operative complications, quantified by univariate logistic regression. Data shown as OR (diamonds) ± 95% CI. Statistically significant associations are shown in red. OR > 1 indicates that the characteristic is associated with a higher-than-baseline risk of complications, while OR < 1 indicates an association with a lower risk of complications. Raw data is available as scatterplots in Supplementary Fig. 7. The figure is available in colour online. Size_mm_12, cyst size > 12 mm; Visual_sx, visual symptoms; Neurology_NOS, neurology not otherwise specified; Resection_extent, cyst resection, as opposed to cyst fenestration; Sleep_fatigue, sleep disturbances or fatigue; Pysch_depression, psychiatric symptoms of depression; Other_sx, any of the following symptoms: ‘Cognitive’, ‘Transient_LoC’, ‘Sleep’, ‘Pysch_depression’, ‘Seizure’, ‘Neurology_NOS’, ‘Fatigue’; LoC, loss of consciousness

The overall complication rate was 17% (47/280) (Fig. 6). The consequences of complications to patients were transient in 13% (36/280) and permanent in 4% (10/277). There was one case of peri-operative mortality due to post-operative myocardial infarction [30]. The frequencies of individual complications are listed in Fig. 6. All complications had resolved by last follow-up apart from 3 cases of visual disturbances, 2 cases of chronic incisional pain, 1 case of sensory disturbance, 1 case of fatigue, 1 case of cervicalgia, 1 case of cerebellar stroke and 1 case of myocardial infarction. More details about each case is available in the legend of Fig. 6. The onset of complications is independent of any predictors except for ‘Cognitive’, which is associated with a lower complication rate. This is explained by the fact that one of the largest case series reports a higher rate of cognitive deficits and a lower rate of complications than the rest of the cohort [12]. The association is no longer significant when this is accounted for.

### Secondary outcomes—subgroup analysis

Adults ($\ge$ 18 years) composed 83% (233/280) of the cohort, with 80% being female. The rate of improvement was 91% and complications rate 16%. All 10 long-term complications recorded occurred in adults. Univariate logistic regression analysis showed that there is an association between cyst size and favourable outcome (OR = 1.22, 95% CI: 1.05–1.41). (Supplementary Table 5). There may also be evidence of an association between the onset of complications and worse outcomes, although this was below the threshold of statistical significance in this retrospective series (OR = 0.44, 0.16–1.21, *p* = 0.11). Both effects were lost in multivariate analysis. On exploratory analysis, we found that the relationship between cyst size and outcome was not linear. ROC analysis revealed a threshold cyst size of 12 mm to be the optimal separator of outcomes within our dataset. Importantly, the cyst size threshold = 12 mm is arbitrary and optimised to this dataset. Its clinical utility is currently very limited, as it is pending external validation on a distinct dataset (Supplementary Fig. 4). There was no additional association between complications and any of the covariates.

Paediatric patients (< 18 years) composed 19% (55/280) of the entire cohort, of which 67% (37/55) were older than 10 years of age. Females constituted 65% of the paediatric cohort, which is significantly less than in the adult cohort (*p* = 0.03). Paediatric patients presented with a lower rate of transient loss of consciousness and focal neurology (Supplementary Table 6). The rate of improvement was comparable to that of adults (96% vs 91%, *p* = 0.27), as was the complication rate (22% vs 17%, *p* = 0.50). All complications were transient in this cohort. Paediatric cases presenting with headache were the majority and had a lower complication rate (OR = 0.11, 0.02–0.67). (Supplementary Table 7).

In terms of surgical approach, 90% of cases were operated by SCIT and 9% by OTT approach. The rate of improvement was not significantly different between the two groups (93% vs 86%, *p* = 0.30), nor was the rate of complications (17% vs 14%, *p* = 0.24). Of the 10 long-term complications, 10 occurred in the SCIT group and 0 in the OTT group. No additional covariate analysed was associated with outcome nor with complications in either subgroup, with the exception of age and cyst size, which were associated with worse (OR = 0.95, 0.91–0.99) and better (OR = 1.20, 1.04–1.40) outcomes, respectively. (Supplementary Table 8).

Despite the large difference in the number of cases managed by cyst resection (*N* = 252) versus fenestration (*N* = 9), the improvement rate for resection is significantly higher than fenestration (OR = 12.64, 3.07–52.01).

## Discussion

We set out to systematically evaluate all existing relevant data on the management of patients with nhSPCs to assist physicians with counselling of their patients regarding the role of surgery. We conducted a rigorous review of published literature and performed meta-analysis of a cohort of 294 surgically treated nhSPCs patients. As the current literature consists of case reports and retrospective series, this review is essentially a summary of all existing retrospective data relating to the management of nhSPCs.

We found that following surgery, 93% of 280 patients with nhSPCs experienced improvement of their symptoms. When only cases with resection are considered the improvement is 94%, while improvement rate is lower where fenestration only was performed (56%). There was remarkably little variation in the rates of improvement among the reported series. All inter-author variability was explained by the number of fenestrations (as opposed to resection) performed. (Supplementary Fig. 3 and Supplementary Table 9). Given the small number of cases treated by fenestration and the number of potential reasons why fenestration only was carried out/achieved in these specific cases, it is best to treat this observation as a hypothesis for future studies rather than a guideline to be used in a clinical practice.

Some authors attempted to objectivise their evaluation by introducing the Chicago Chiari Outcome Scale [7, 13, 28], the EORTC QLQ-C30 [14] or their own bespoke symptom scoring [10, 12]. However, in most cases, the overall surgical outcome was mostly derived from clinical consultations when patients would be asked whether they are overall better or worse as a result of the operation. As much as these statements seem to express what really matters to patients, they are associated with a risk of bias stemming from patient reporting directly to the treating surgeon who in turn is recording these. Therefore, more objective measurements of quality of life need to be agreed upon and prospectively collected to provide more solid evidence regarding the benefit of surgery in the management of nhSPCs patients.

When considering each symptom individually, more than 85% of patients experienced improvement in most symptoms, with the exception of cognitive deficit, fatigue and sleep disturbance, which improved in 80%, 68% and 45% of patients, respectively (Supplementary Table 10). This is not surprising as the latter three symptoms have complex multifactorial underpinning. It is noteworthy that symptoms with perhaps the most tenuous etiological link to the pineal cyst, such as episodic loss of consciousness, seizures and psychiatric symptoms, improved in 97%, 100% and 100%, respectively. Headaches, the most common symptom, improved in 93% of patients. This has previously been shown in a systematic review focused on headaches [31].

Complications occurred in 17% the 280 surgically treated cases. Most of the impairment resulting from complications resolved by the last follow-up, while 10 patients (3.6%) experienced permanent adverse effects of surgery. One patient died post-operatively secondary to myocardial infarction [30]. Although the mortality seems unrelated to the surgery itself, it is a stark reminder that any surgical intervention comes with risks of morbidity and mortality against which the decision to operate must always be carefully considered.

**Fig. 7:**
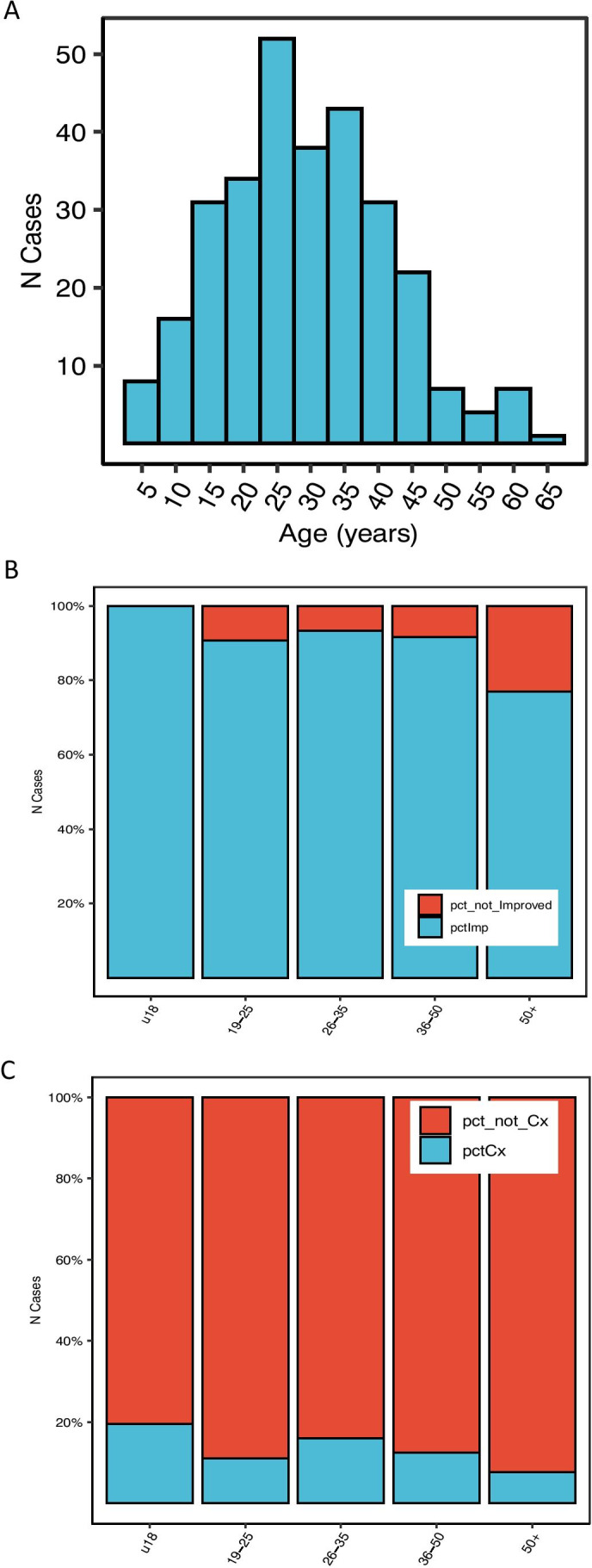
Summary of the effects of age on safety and efficacy of nhSPC surgery. **A** Age distribution across the cohort. **B** Proportion of improved patients for each age group. **C** Proportion of cases with complications in each age group. The figure is available in colour online

Comparable rates of improvement and complication rates following surgery were observed in adult and paediatric (< 18 years) patients (91% vs 96% and 16% vs 22% respectively). Similar results (96% improvement, *N* = 109) were reported in a systematic review of patients younger than 21 years by Choque-Velasquez et al. [6]. This is not surprising as much of the data in this paper and the current study overlap, although different age definitions and methodologies were used. Ages of patients in this cohort were normally distributed with a mean and SD of 29.3 and 12.3, respectively (Fig. 7). Interestingly, the improvement rate of patients older than 55 years is lower than that of younger patients (OR = 0.11, 0.02–0.79, *p*. val = 0.01). This may suggest that other factors, such as perimenopausal changes, may play a role in the aetiology of at least some symptoms in this subset of patients. Interestingly, the complication rate may be lower in this age group (not significant but underpowered, 12% vs 17%).

The majority of resections were caried out using SCIT, while a few using OTT. There was no difference in outcomes in patients treated by either approach. The choice of approach was likely a matter of greater familiarity, but also individual anatomical considerations likely played a role [46]. Based on the data from our meta-analysis, it seems that resection is more effective than fenestration (OR = 12.64; 3.07–52.01). Other means of surgical treatment of patients with nhSPC have been described. Davidson reports his experience with endoscopic management of 16 patients with ‘pineal cyst-associated aquaeductal stenosis’, where 10 of the patients had no ventriculomegaly so, technically, could be classed as nhSPCs [9]. All these 10 cases were treated with endoscopic third ventriculostomy (ETV) and 7 of these patients improved (mean follow-up 10 months). Interestingly, Eide et al. treated 6 patients with insertion of ventriculoperitoneal shunt, but only one patient had improved [12].

There is very little published on conservative management of patients with nhSPCs, and the data assembled in this meta-analysis cohort (*N* = 80) are probably most informative on the subject. Despite, best non-surgical management symptoms did not improve in 87% and worsened in 13% of patients during a mean of 52 months of non-surgical management. These data are broadly accordant with the conservatively managed patients in the series by Eide et al. [10] and Majovsky et al. [28]. In the former series (*N* = 66, mean follow-up 3 years), 11% improved, 14% were unchanged and 75% worsened while in the latter (*N* = 110, 6.5 years), 12% improved, 74% were stable and 16% worsened. Given the lack of strict prospectively adopted definitions of the conservative management, selection bias and other shortcomings, these cohorts are not suitable as direct controls for the surgical group. Importantly, before considering surgical management of patients with suspected nhSPCs, it is essential to exclude other causes of patients’ symptoms as highlighted in all published series (see also Fig. 2).

It is the authors’ experience that understanding the subtleties of presentation of patients with nhSPC is of crucial importance in the elicitation and recognition of all existing symptoms. Despite the retrospective nature of the input data and with its associated varying levels of detail about presenting symptoms—both between published studies and individual cases within each study—this metanalysis was fruitful in providing the most comprehensive description so far of the characteristics of nhSPC as a disease entity. This said, it is important to recognise that, overall, these data are reductive. Below, we briefly share our observations of the most common symptoms and clinical phenomena and suggest potential links to their aetiologies. As much as we hope that the reader may find this helpful, the validity of these observations needs to be explored in prospective studies.

Headaches are the most common and usually the dominant symptom, yet probably the least well understood. It is our experience that headaches associated with nhSPCs have often more than one component: one commonly described as constant dull pressure or fullness and the other ‘migrainous’. Indeed, not infrequently patients are treated for migraines with only partial or no success. As shown in this paper, female patients make up 80% of adult nhSPC patients, while only 65% of patients below 18 years of age. Like migraine headaches, headaches in nhSPC patients can be associated with the menstrual cycle. It is therefore likely that hormonal changes directly and/or through body fluid content and its redistribution play a role in the aetiology of headaches and other symptoms of nhSCP patients. Typically, the ‘non-migraine headaches’ resolve following surgery, while the ‘migraine headaches’ either also resolve or, if not, the frequency and duration of migraine attacks almost always abate.

Visual symptoms are often reported as blurred vision, double vision, delayed acquisition or binocular fusion of visual images after gaze change or simply as ‘tired’ and painful eyes. Prolonged work on a computer or frequent switching of gaze, such as driving a car, especially at night, can trigger headaches, vertigo and disorientation. Bedside eye examination is usually normal, although some limitations or discomfort on gazing upward is not uncommon. Some or all components of Parinaud’s syndrome can sometimes be demonstrated. Ophthalmological examination usually fails to demonstrate any additional abnormalities, but more specialised pursuit examinations are rarely carried out. These dorsal midbrain symptoms are likely a result of direct compression by the cyst.

Other symptoms. The tectum, especially the superior colliculus, is not only important in processing visual and auditory information, but is also a centre of multimodal sensory integration involving visual, auditory, vestibular and other somatic sensory information [5, 19, 25, 26, 35, 37–39, 41]. More recently, superior colliculi have also been linked with cognition [3, 22]. Indeed, ‘headache-visual’, ‘headache-visual-nausea/vomiting’, ‘headache-visual-vertigo/dizziness-neurology not otherwise specified’ were most common when combinations of two, three and four symptoms were considered (Fig. 4 and Supplementary Fig. 5). It is reasonable that visual and balance-associated symptoms, such as dizziness, vertigo, unsteady gait and nausea can be attributed to interference of the pineal cyst with tectal processing. It is also possible that other symptoms otherwise difficult to explain, such as sensory symptoms (e.g. ‘neurology_NOS’ in our series) and even some ‘psychiatric’ symptoms (e.g. multisensory dysfunction and dissociative disorders) could be explained by a similar mechanism [34]. Presenting symptoms should not outright be dismissed on the basis of lack of understanding of their aetiology. In fact, given the improvement of ‘psychiatric symptoms’ following surgery in nearly 100% of patients in this relatively small cohort (Supplementary Table 10), one should keep an open yet critical mind. As pointed out by Majovsky et al. [28], certain symptoms are likely the result of ‘somatisation’, e.g. secondary to chronic headaches, sleep disturbance, difficulty with fulfilling personal and family, work and other wider social expectations. This can be further compounded by lack of effective treatment and consequent feeling of hopelessness.

Patients’ symptoms sometimes worsen during pregnancy, after gaining weight and are often worse in the morning. Some patients require an hour or more after getting up for their symptoms to subside sufficiently for them able to function. They often describe this as a ‘bad hangover’. These factors suggest CSF/venous aetiology. Interestingly, intracranial pressure (ICP) is generally not raised in patients with nhSPCs, and ICP monitoring studies are not routinely undertaken. Eide and colleagues studied overnight ICP parameters in 20 nhSPCs and compared them with that of 19 patients with chronic daily headaches (CDH), i.e. patient suspected of idiopathic intracranial hypertension without papilloedema [11]. Both groups had relatively normal mean static ICP, while nhSPC patients had higher pulsatile ICP scores than CDH patients. Six were treated with a ventriculoperitoneal shunt (VPS) and 14 underwent resection of their pineal cyst [12]. While none of the ICP parameters differed significantly between the VPS and resection groups, patients treated with resection enjoyed significantly greater improvement of symptoms. These results suggest that symptoms in most nhSPC patients are not determined by globally raised ICP, but more subtle and probably more localised effects of the pineal cyst; likely, a result of a combination of the direct compression of the tectal plate and crowding the quadrigeminal cistern, thus, preventing sufficiently effective opening of the aqueduct during systole as well as interfering with the deep venous flow during both, systole and diastole [12]. Overall, the aetiology of the symptoms is not well understood, and this major shortcoming needs to be addressed in future studies.

Placebo effect is sometimes suggested as the reason behind the improvement of symptoms following surgery in patients with nhSPCs. It is true that undergoing brain surgery is a profound experience for patients, but placebo alone is unlikely to be effective in such a high proportion of patients (93%), and it is even less likely that the effect would persist at a mean of 34.6-month follow-up. The observation of poorer symptom control following cyst fenestration compared to resection (OR = 12.64, *p* = 0.0004) also argues against placebo, although it is not possible to rule out the influence of patient interpretation of their post-operative scans as ‘the cause of my problem is no longer there’ versus ‘it is still there’. Furthermore, despite the heterogeneity of cultural background, clinical practice and the level of detail in recording and reporting of clinical data, the review has uncovered remarkable concordance in the main baseline clinical characteristics and outcome between the included studies (see Figs. 4 and 5).

While this meta-analysis has been carried out with a great deal of scientific rigour, the results must be interpreted with caution. One must keep in mind the difference between internal validity, which depends on the quality of the analysis, and external validity, which is a function of the quality of the data.

The conclusions taken from the input data are internally valid, in that they describe properties of the dataset analysed in a scientifically rigorous fashion. However, their external validity (i.e. their applicability outside of this dataset, into the real world) is limited by the quality of the data from which they originate. Given that the input data are derived from case reports and retrospective, single-surgeon cohort studies, they are inherently associated with several of limitations. These include (1) Incompleteness and heterogeneity of data collection and reporting. This applies especially to the publication bias but also the inevitable lapses in detection and recording of post-operative complications. We tried to mitigate the publication bias, i.e. a greater likelihood of reporting cases with favourable outcome, by only considering consecutive series when calculating safety and efficacy. Despite of this, it is likely that the rate of long-term complications is underestimated. (2) Lack of objective definition of symptoms and outcome measure. This is further compounded by a potential bias related to patients reporting outcome to their treating surgeons and the treating surgeons recording these outcomes in the majority of cases. (3) Lack of appropriate control of conservatively managed cohort.

Future work needs to address these limitations by defining, objectivising and standardising assessment of presentation and outcome, both in terms of symptom and quality of life. In addition, systematic mapping and evaluation of non-surgical treatment needs to be caried out. Prospective studies with these carefully defined clinical data points will provide an important knowledge base for recognising patients with nhSPCs and estimation of the likelihood for improvement of each symptoms and overall quality of life of each patient at individual level. Authors of this study are proposing the formation of an international registry for this purpose. A randomised controlled trial of conservative versus surgical treatment would provide ultimate answers regarding the safety and efficacy of surgical treatment of nhSPCs. Design and execution of such as trial is associated with numerous potential challenges, including the reluctance of patients to be randomised, selection criteria for participating centres etc. Lastly, although several hypotheses about the link between PCs and symptoms have been put forward, there is very little scientific evidence to back or disprove these. Employment and thoughtful analysis of the existing and novel imaging techniques, computerised ICP measurements and, possibly, intraluminal venous pressure studies as well as systematic translation of the results of animal neuro-physiology to humans will be required to fill these knowledge gaps. This will enhance the objectivity and accuracy of assessment, selection of appropriate treatment and, ultimately, contribute to improvement of quality of life of patients.

## Conclusion

This meta-analysis summarises current evidence on the role of surgery in the management of nhSPCs. Of the 280 patients from 19 consecutive series treated surgically for nhSPCs, 93% reported overall improvement. Complication rate was 17%. There was one case of peri-operative mortality, and permanent complication-related morbidity occurred in 10 cases (3.6%). This meta-analysis was carried out with high level of scientific rigour, and conclusions derived from the analysed dataset are internally valid. However, the external validity, i.e. their applicability outside of this dataset, is constrained by limitations of the input data. Therefore, a great deal of caution must be exercised when interpreting and applying the findings to clinical practice. The authors hope that this manuscript will provide the foundation to the design of rigorous prospective studies to quantify safety and efficacy of this intervention.

## Supplementary Information

Below is the link to the electronic supplementary material.

Supplementary file1 (PDF 1090 KB)

Supplementary file2 (XLSX 115 KB)
