# Supplementary material for: Surgical treatment of symptomatic pineal cysts without hydrocephalus—meta-analysis of the published literature
Source: Acta Neurochir (Wien). 2021 Dec 2;164(1):61–77. doi: 10.1007/s00701-021-05054-0 (PMC8761144; doi:10.1007/s00701-021-05054-0)
Supplement: Supplementary file 1 — Supplementary file1 (PDF 1090 KB) [file 701_2021_5054_MOESM1_ESM.pdf]

Supplementary Figures:

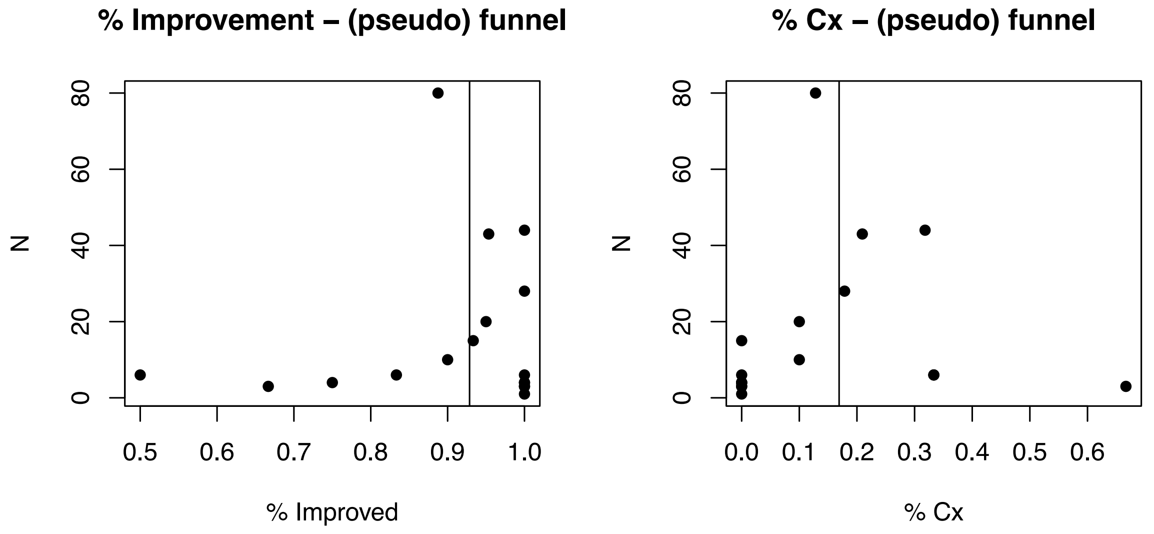

Supplementary Fig. 1. Visualization of possible reporting bias.
Cx= Complications

Supplementary Fig. 2. Summary of the quality of evidence that met both inclusion and exclusion criteria. Quality of evidence matrix. Questions are listed below.

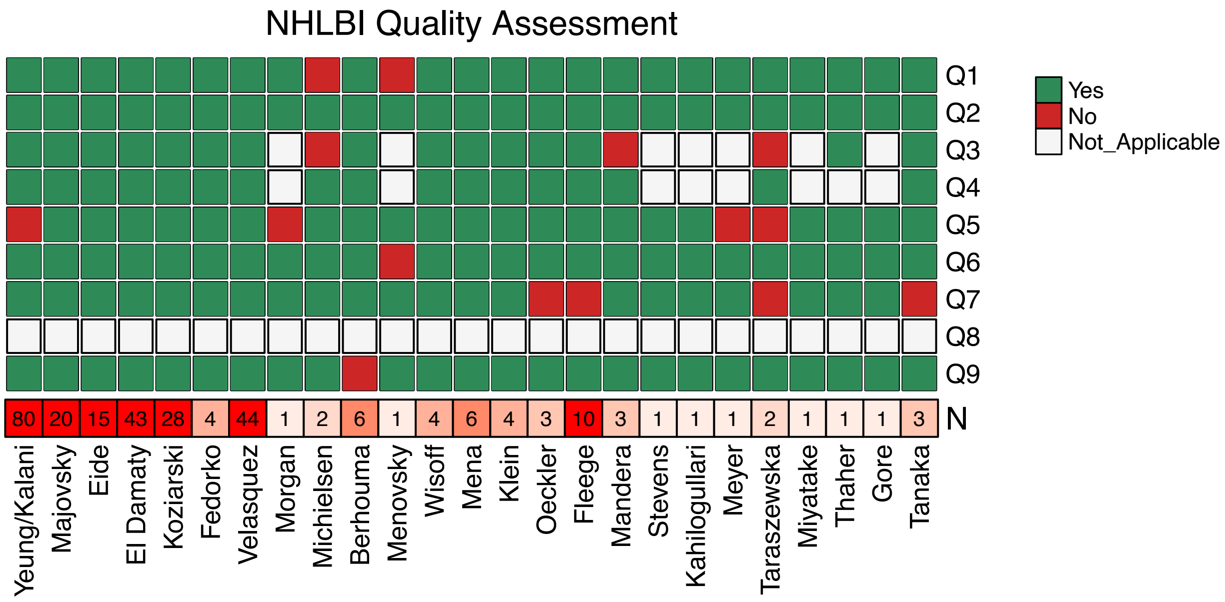

Q1: Was the study question objective and clearly stated?
Q2: Was the study population clearly and fully described, including a case definition?
Q3: Were the cases consecutive?
Q4: Were the subjects comparable?
Q5: Was the intervention clearly described?
Q6: Were the outcomes measures clearly defined, valid, reliable, and implemented consistently across all study participants?
Q7: Was the length of follow-up adequate?
Q8: Were the statistical methods well-described?
Q9: Were the results well-described?
The figure is available in color online.

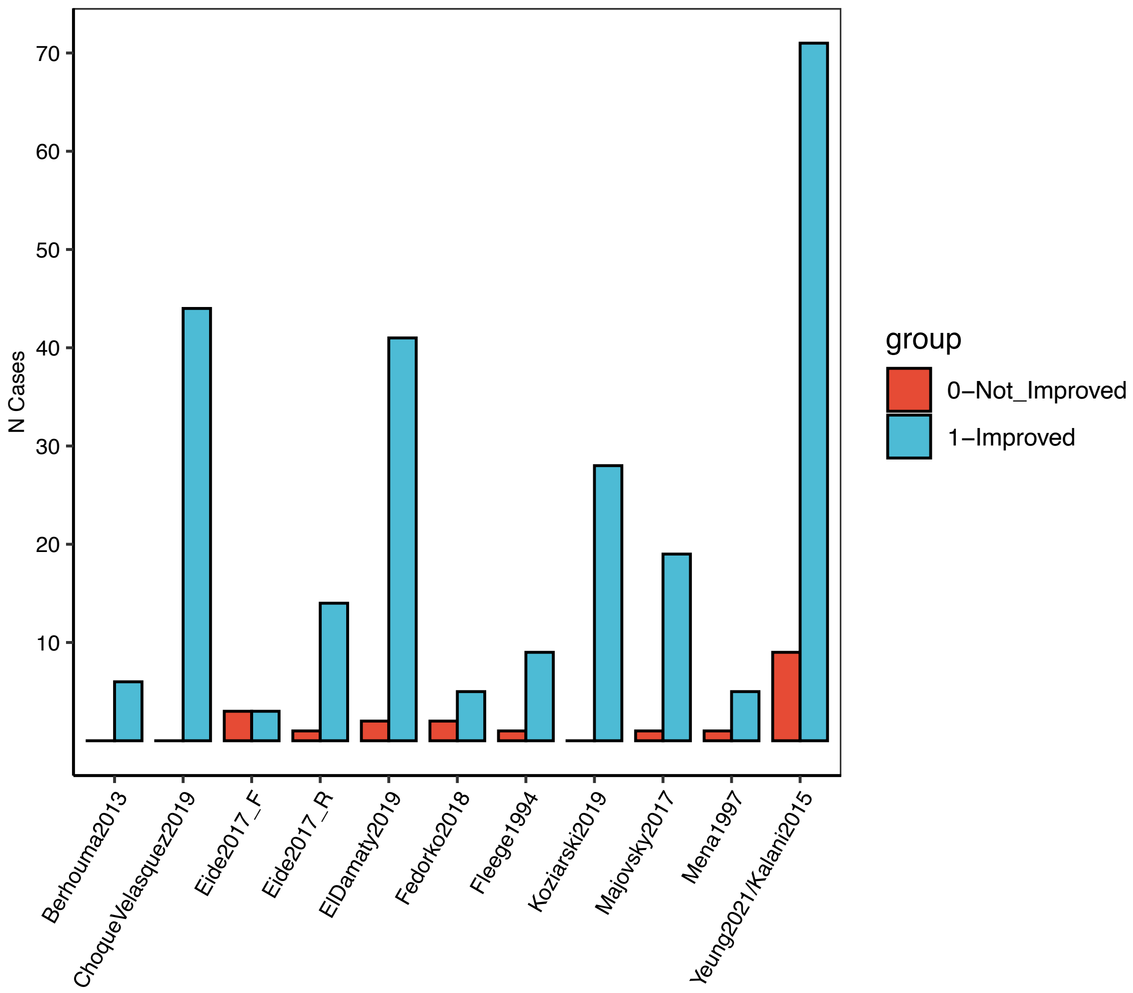

Supplementary Fig. 3. Supplementary Figure to show the comparison between the reported improvement rate between different authors. Only authors with N>5 are shown.
Refer to Supplementary Table 9 for a summary of the statistical analysis and a brief discussion of salient findings.

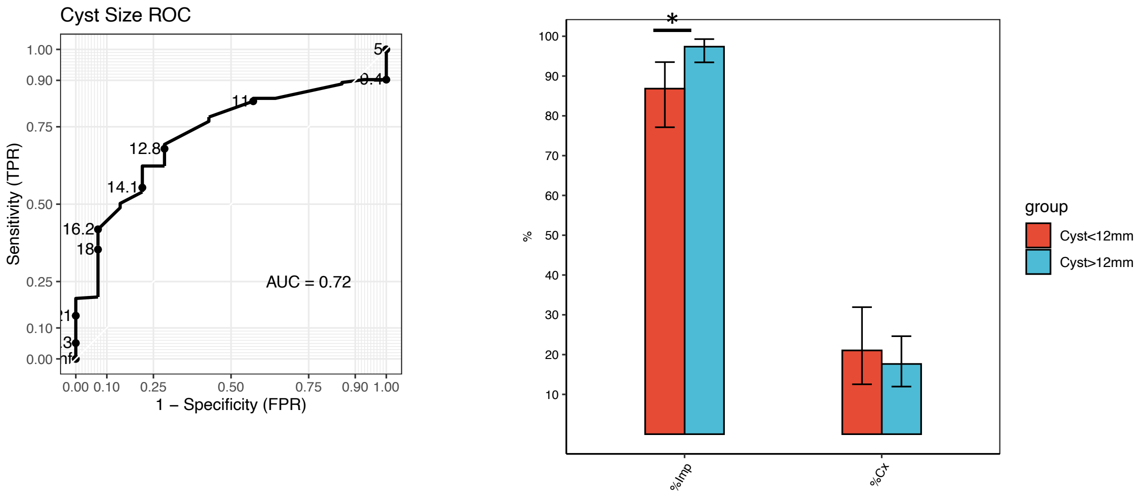

Supplementary Fig. 4. Cyst size threshold optimization. From the scatterplot in Supplementary Fig. 5 it appears that the relationship between cyst size and outcome is not linear. Exploratory post-hoc analysis identified an optimal threshold of CS=12mm able to best discriminate outcomes. Of note, cyst size has no effect on complication rate neither as continuous nor categorical variable.

NB: The cyst size threshold =12mm is arbitrary and optimized to this dataset. Its clinical utility is therefore very limited, pending its validation on a distinct dataset.

Supplementary Fig. 5. Supplementary Figure showing the number of patients presenting with each combination of 1, 2, 3, 4, 5+ symptoms (left to right, respectively)

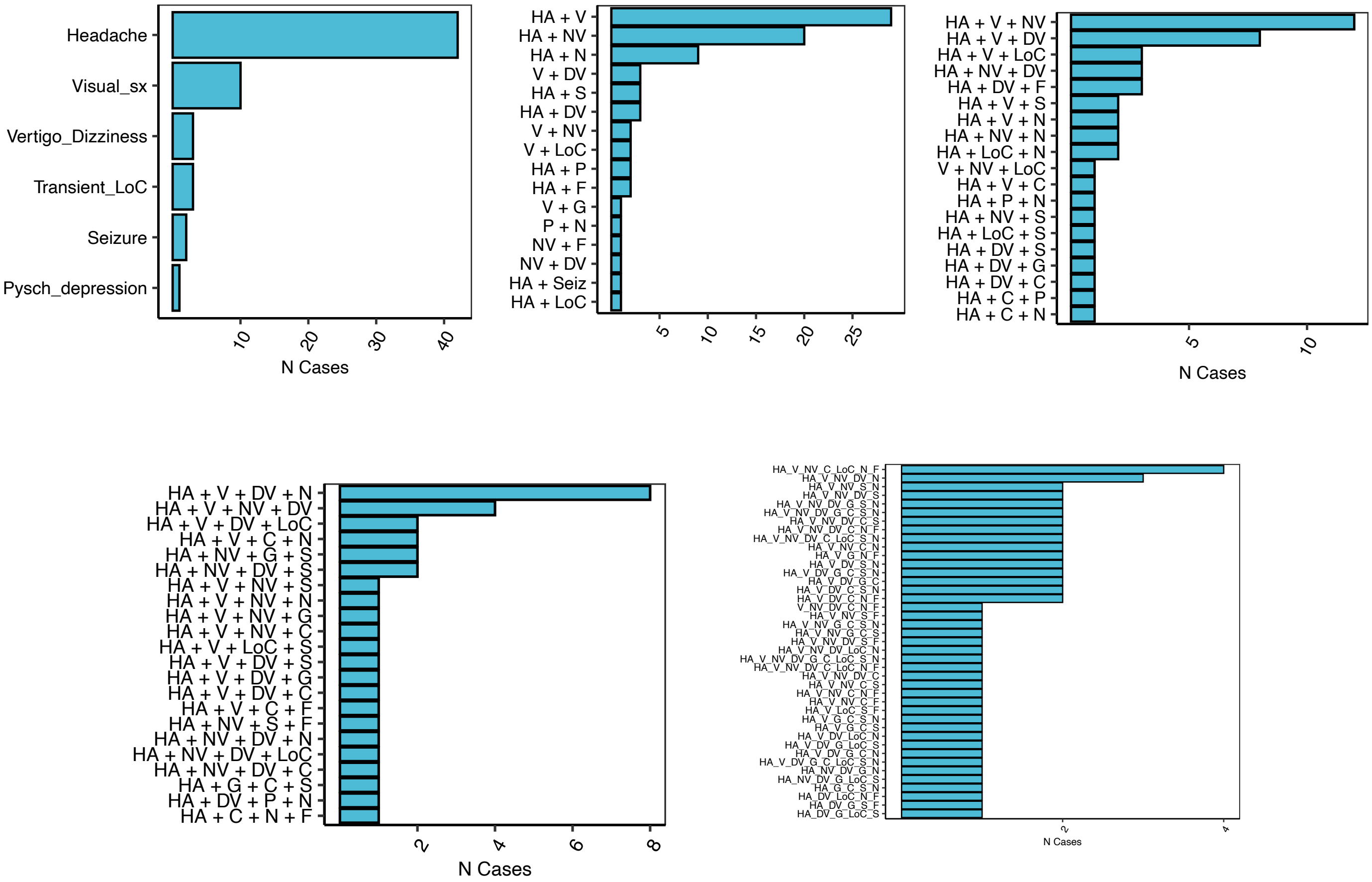

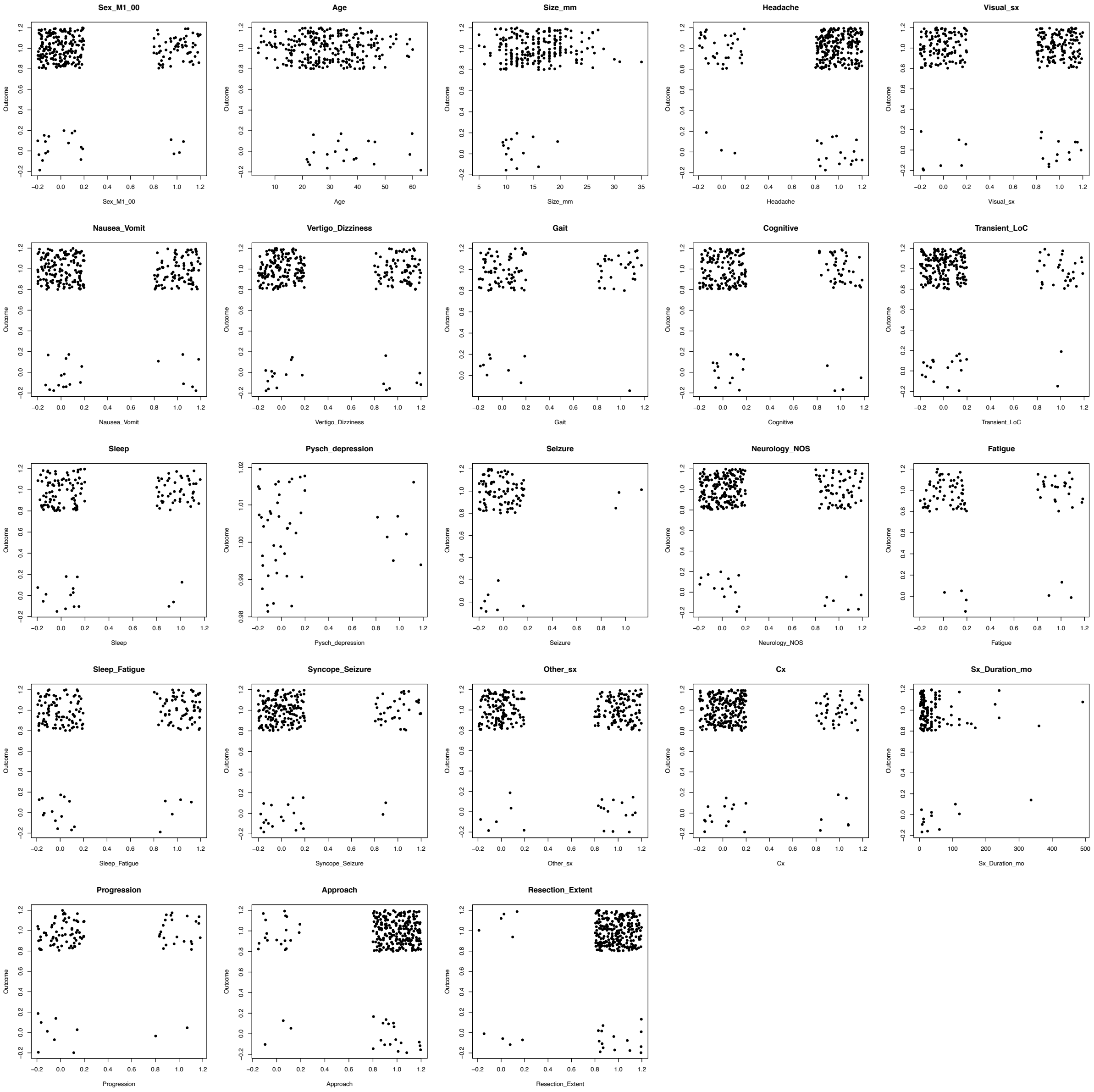

Supplementary Fig. 6. Supplementary Figure to show the relationship between each characteristics and patient outcome. Outcome=1 indicates improvement, whilst Outcome=0 indicates no improvement. For categorical variables x and y jitter has been introduced to generates density clouds. For continuous variable jitter was only applied to the y axis.

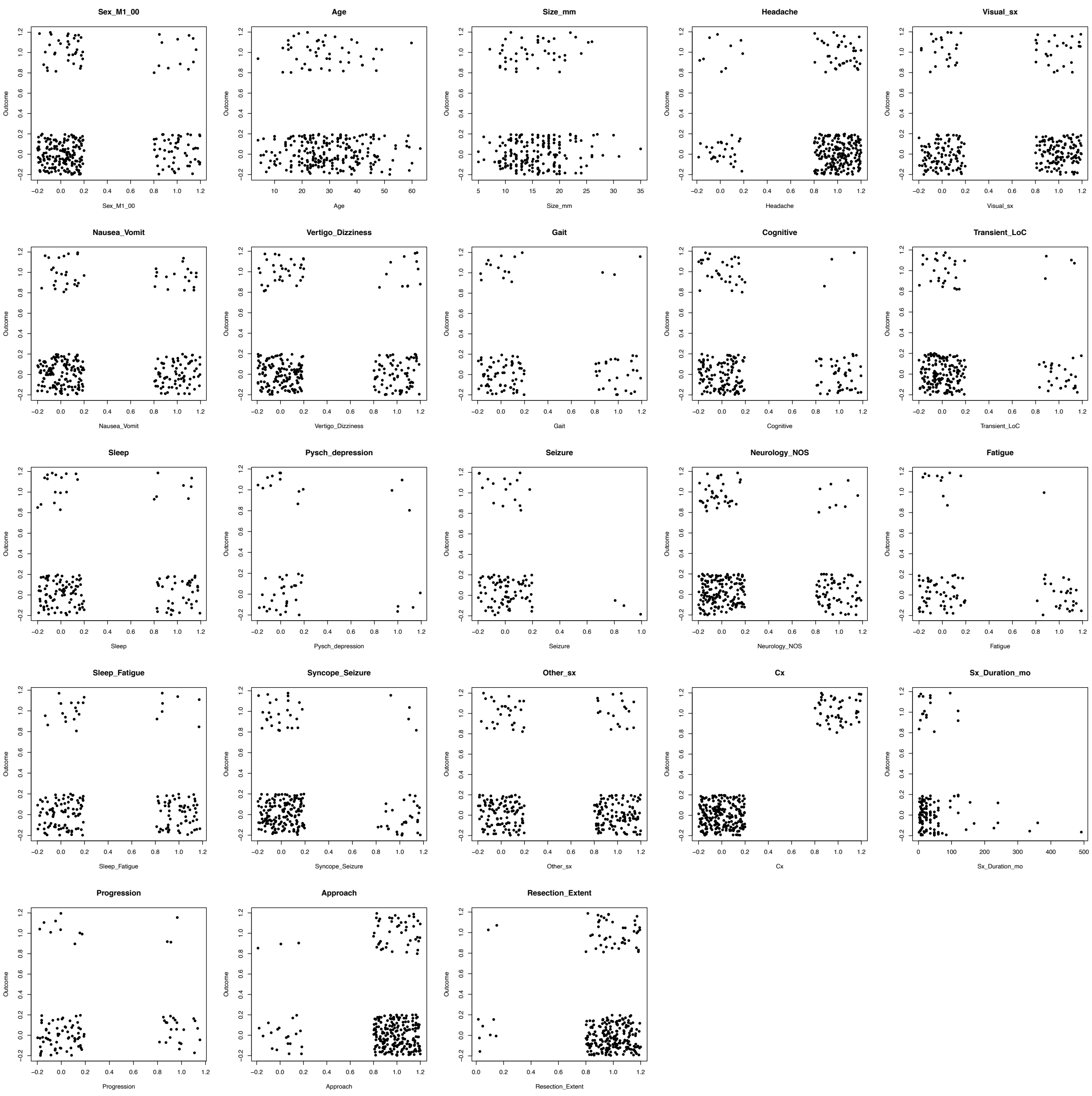

Supplementary Fig. 7. Supplementary Figure to show the relationship between each characteristics and patient complications. Outcome=1 indicates complications, whilst Outcome=0 indicates no complications. For categorical variables x and y jitter has been introduced to generates density clouds. For continuous variable jitter was only applied to the y axis.
